# Multidimensional molecular strategies for crop photoperiod adaptation and latitudinal expansion

**DOI:** 10.3389/fpls.2026.1967465

**Published:** 2026-10-12

**Authors:** Hang Zhao, Shuxin Zhang, Xiaoyu Yin, Nianwei Qiu, Ying Bao

**Affiliations:** 1 School of Life Sciences, Qufu Normal University, Qufu, Shandong, China; 2 Shandong Key Laboratory of Wetland Ecology and Biodiversity Conservation in the Lower Yellow River, Qufu, Shandong, China

**Keywords:** crops, flowering, latitudinal expansion, photoperiod, reproductive

## Abstract

The traditional regional distribution of agriculture was historically constrained by crops’ inherent dependence on local environmental cues, particularly photoperiod and temperature. Natural domestication and artificial selection have progressively reduced this dependence on native ecosystems, resulting in a profound restructuring of global agricultural patterns. From the perspective of this global restructuring, this study systematically examines the multidimensional molecular mechanisms underlying photoperiodic adaptive evolution, including population-level point mutations, epigenetic modifications, structural variations, and copy number variations. In parallel, we propose that the latitudinal adaptive expansion of crops represents not merely an ecological niche shift, but a complex evolutionary process driven by coordinated genetic reprogramming across the genome, epigenome, and transcriptome. Consequently, reshaping future agricultural landscapes requires moving beyond the constraints imposed by individual climatic factors and establishing a multidimensional framework that integrates environment, genetic traits, and ecological adaptation. Moreover, rather than focusing solely on the speed of regional expansion, future molecular design should promote the coordinated improvement of broad-spectrum latitudinal adaptability and elite yield-quality traits, thereby securing global food safety and agricultural sustainability.

## 1 Introduction

The continued expansion of the global population, together with the rising frequency of extreme weather events, has placed considerable pressure on efforts to ensure food security and maintain a stable supply of essential agricultural products (Godfray et al., 2010). During natural evolution and early artificial domestication, crops acquired strong adaptability to the climatic conditions prevailing in their regions of origin (Meyer and Purugganan, 2013). However, as arable land resources become increasingly scarce, the introduction and expansion of crops from their original habitats into other latitudinal zones have become an unavoidable trend in modern agricultural development. During crop range expansion, environmental factors such as photoperiod, which regulate flowering time, function as major constraints (Jung and Müller, 2009; Nakamichi, 2015). When crops are introduced into new latitudes, changes in external light signals can induce abnormal flowering (e.g., delayed or premature onset) and restrict biomass accumulation, ultimately resulting in substantial yield losses. Therefore, overcoming inherent photoperiod constraints and improving environmental adaptability are essential for successful cross-regional introduction and expansion, as well as for increasing crop yields.

The latitudinal expansion of crops represents more than a geographical shift; it is a complex evolutionary process involving coordinated reprogramming across genomic, epigenomic and transcriptomic layers (Hufford et al., 2012; Liu et al., 2020). However, a unified theoretical framework connecting multi-scale evolutionary drivers with contemporary molecular strategies remains notably lacking. Consequently, research in this field remains fragmented across individual scales or isolated trait modifications and does not fully address the integrated breeding objectives required for pyramiding cross-latitude adaptive traits. Accordingly, we examine the multidimensional adaptive strategies that underlie range expansion, including population-level point mutations, epigenetic modifications, structural variations, and copy number variations. Furthermore, we discuss the challenges associated with reshaping future agricultural landscapes from the perspectives of multi-level genetic information reprogramming and the coordinated optimization of adaptive expansion while retaining superior traits.

## 2 Tracing the discovery and breeding trajectories of photoperiodism in crops

Crops geographic distribution and adaptive range expansion are closely tied to photoperiodic responses. Scientific inquiry into this phenomenon dates back to the early 20th century, when W. W. Garner and H. A. Allard discovered that tobacco flowering is regulated by seasonal day length. They observed that while tobacco plants remained vegetative in summer, they flowered normally in winter greenhouses. Intriguingly, artificially shortening the daytime length in summer accelerated flowering, whereas extending winter photoperiods inhibited it. This physiological mechanism, by which plants perceive external light cues to anticipate seasonal transitions and adjust their development accordingly, came to be known as photoperiodism. Based on their responses to day length, plants are generally classified into three categories: short-day, long-day, and day-neutral plants (Amasino, 2013; Garner and Allard, 1920) (**Figure 1**).

**Figure 1:**
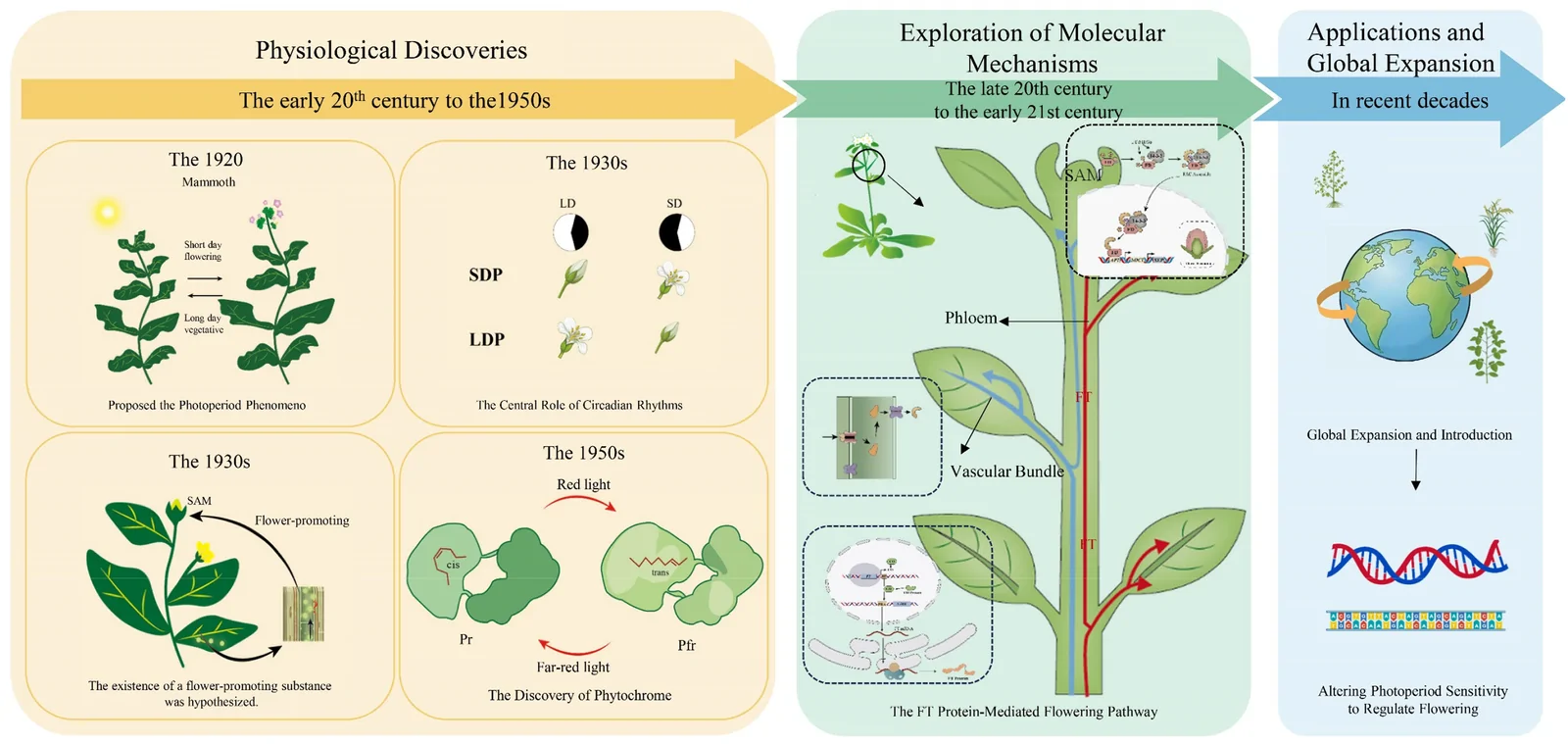
Major evolutionary milestones in crop photoperiodic research. The timeline illustrates the systematic research progression understanding of photoperiodism, advancing from early phenotypic observations and in-depth molecular mechanism analysis to modern agricultural applications. The first stage, the discovery of photoperiodism spanning from the early 20th century to the 1950s, integrates the initial proposal of the photoperiod phenomenon in the 1920s, the establishment of the core role of circadian rhythms and the florigen hypothesis in the 1930s, and the discovery of phytochrome in the 1950s. The second stage, the exploration of molecular mechanisms from the late 20th to the early 21st century, focuses on revealing the flowering signaling pathway centered on the FT (florigen) protein, along with the molecular network governing its transport from leaves through the vascular phloem to the shoot apical meristem (SAM). The third stage, the application of photoperiodism in global expansion over recent decades, involves precisely modifying crop photoperiod sensitivity to regulate flowering time, ultimately reshaping the global latitudinal distribution patterns of crops.

Subsequent studies identified leaves as the primary organs responsible for perceiving photoperiodic signals, leading to the proposal of a mobile floral stimulus termed “florigen”, which moves through vascular tissues to the shoot apex to induce flowering (Chailakhyan, 1937). With the rapid progress of molecular biology and genetics, research on crop photoperiodism has transitioned from macroscopic observations to in-depth mechanistic exploration at the molecular level. In 2007, the FT protein was finally confirmed as the elusive florigen. Under inductive photoperiods, *FT* is expressed in leaf phloem companion cells, and the synthesized FT protein is then transported through the phloem to the shoot apical meristem, where it assembles florigen activation complex (FAC) with FD and 14-3-3 proteins, ultimately triggering the floral transition (Corbesier et al., 2007; Gao et al., 2026, 2025; Zhao et al., 2026) (**Figure 1**).

Recent advances in population genetics have made it possible to trace crop evolutionary trajectories during domestication and range expansion. Leveraging advanced approaches such as genome-wide association studies (GWAS) and gene recombination technologies, key regulatory genes governing photoperiodic responses in crops have been identified, along with critical natural variants and point mutations that lead to loss-of-function or enhanced/weakened activity of these genes. These genetic alterations have modified the intrinsic photoperiod sensitivity of crops, thereby effectively removing the geographical constraints that once limited their transregional expansion. As a result, large-scale introduction and substantial yield enhancement have been achieved worldwide—a trend particularly notable in cotton, which has undergone a remarkable transition from a photoperiod-sensitive wild type to a day-neutral cultivated form (Andrés and Coupland, 2012).

## 3 Multidimensional molecular evolutionary dynamics in reshaping crop photoperiod adaptation

The adaptation of crops to photoperiods and their global expansion across different regions are not dependent on a single genetic strategy. Rather, they are associated with a multidimensional synergistic approach involving genomic structural variations (including population-level point mutations and large-scale genomic variations) as well as epigenetic modifications. Ultimately, these mechanisms break the original photoperiod constraints by reshaping the floral induction signaling pathways, thereby enabling crops to better adapt to diverse ecological environments across different latitudes worldwide (**Figures 2**; **Table 1**).

**Figure 2:**
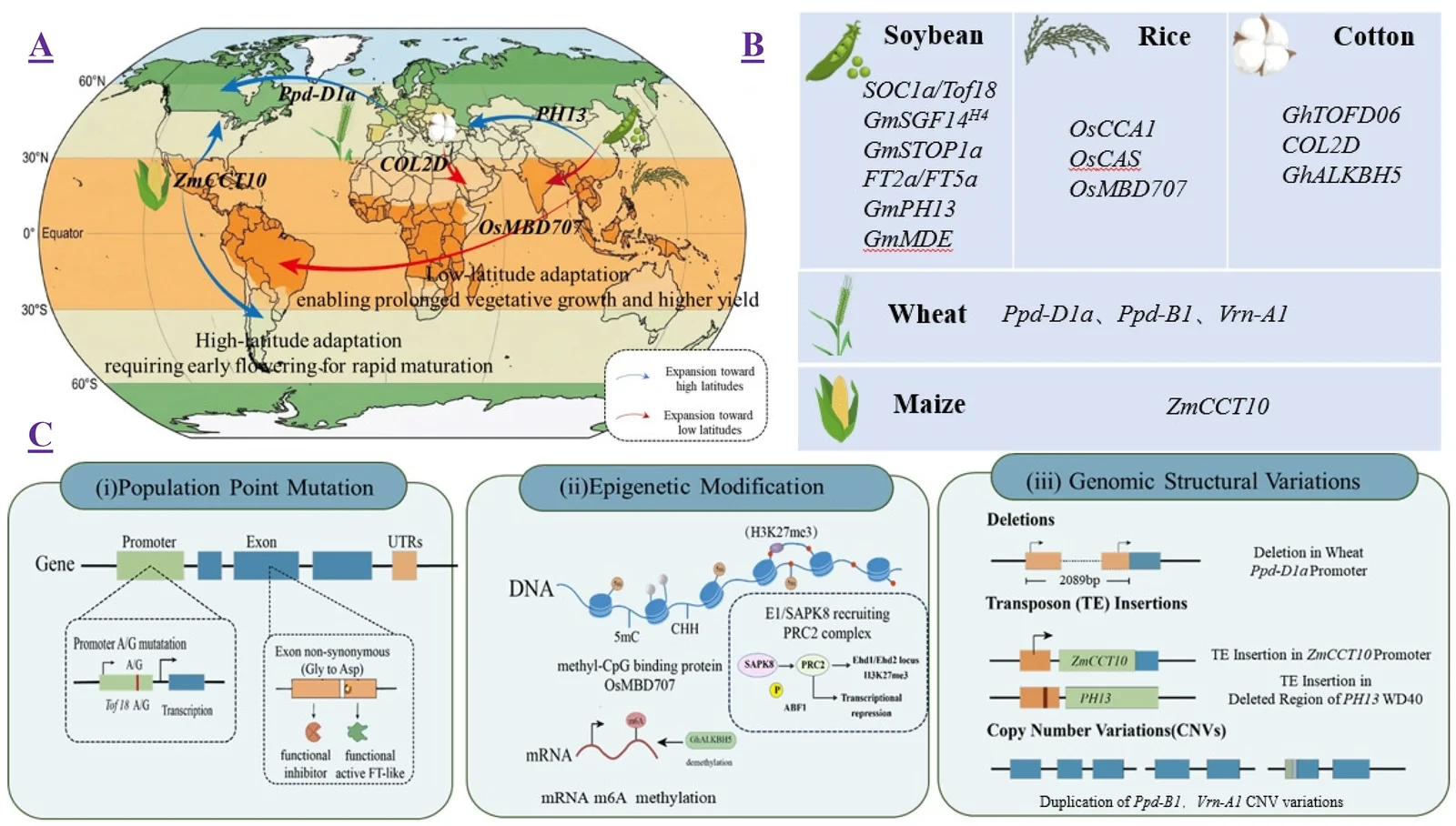
Multidimensional drivers of reshaping the global agricultural landscape. **(A)** Schematic diagram illustrating crop expansion toward high latitudes (blue arrows) and low latitudes (red arrows), driven by key flowering genes. Interactions between key flowering genes and the environment drive crop expansion into different latitudinal regions. **(B)** Key genes regulating photoperiod sensitivity in soybean, rice, cotton, wheat, and maize. **(C)** Multidimensional regulatory mechanisms underlying crop photoperiod adaptation, encompassing point mutations at the population level, epigenetic modifications, as well as structural variations and copy number variations.

**Table 1:** Functional roles of environmental adaptation-related factors in crops.

Crop	Gene/locus	Variant type	Molecular mechanism	Phenotypic effect	Evidence for geographic/latitudinal adaptation	Evidence type	Reference
Soybean	*Tof18/SOC1a*	Promoter A/G point mutation	Divergent transcriptional activity	Tof18^G^ promotes early flowering; Tof18^A^ delays flowering	Tof18^G^ adapts to high latitudes; Tof18^A^ adapts to low latitudes	Association & Functional evidence	(Kou et al., 2022)
Soybean	*GmSGF14^H4^*	Frameshift mutation	Disrupts the core 14-3–3 structural domain	Results in a late-flowering phenotype	Enhances soybean adaptability to specific low latitudes	Association evidence	(Jiang et al., 2023)
Soybean	*GmFT2a*	Non-synonymous mutation in exon	Suppresses its repressor function	Delayed flowering under long-day and high-temperature conditions	Adaptation to specific regional environments	Association & Functional evidence	(Sun et al., 2019)
Soybean	*GmFT5a/qDTF-J*	Non-coding region polymorphisms	Alters transcript abundance independently of E1	Promotes early flowering	Provides genetic basis for early-flowering in high-latitude regions	Association evidence	(Takeshima et al., 2016)
Soybean	*GmSTOP1a*	24-bp InDel in promoter	Disrupts/maintains *GmAGL12* mediated transcriptional activation	Regulates low-phosphorus tolerance	Enhances adaptation to acidic, phosphorus-deficient soils at low latitudes	Direct & Association evidence	(Guo et al., 2026)
Soybean	*GmPH13*	Transposon insertion (WD40 deletion)	Reduces interaction with GmCOP1	Generates shorter internodes and dwarf plants	Enhances yield and shade traits for high-density planting at high latitudes	Association & Functional evidence	(Qin et al., 2023)
Soybean	*GmMDE*	Epigenetic (H3K27me3 modification)	E1 directly elevates H3K27me3 to suppress GmMDE	Coordinates flowering time and termination of shoot apical growth	Potential basis for latitudinal adaptation	Indirect & Functional evidence	(Zhai et al., 2022)
Rice	*OsCCA1*	SNPs in coding region	Functional deficiency induces OsPRR37 and DTH8	OsCCA1a promotes flowering; OsCCA1d delays flowering	OsCCA1a enables expansion to higher latitudes; OsCCA1d stabilizes yields in low latitudes	Association & Functional evidence	(Lee et al., 2022)
Rice	*OsCAS*	Deletion	Modulates sugar partitioning	Induces male semi-sterility	Fertility adaptation in response to varying photoperiods	Indirect & Functional evidence	(Wang et al., 2021)
Rice	*OsMBD707*	Epigenetic/Overexpression	Nuclear-localized MBD protein regulating flowering regulator expression in the Ehd1-Hd3a/RFT1 pathway	Larger tiller angles and reduced photoperiod sensitivity (delayed flowering under SD and early flowering under LD)	Involved in epigenetic regulation of photoperiod response and development in rice	Functional evidence	(Qu et al., 2021)
Cotton	*GhTOFD06*	Non-synonymous mutation in coding region	Attenuates negative regulatory effect on flowering	Reduces photoperiod sensitivity, early maturity	Positive selection during cultivation drives high-latitude adaptation	Association & Functional evidence	(Xu et al., 2026)
Cotton	*COL2D*	Epigenetic (DNA Methylation)	Hypermethylated in wild; demethylated in domesticated	Wild is photoperiod sensitive; domesticated is insensitive	Broadens cultivation latitude and environmental resilience	Association & Functional evidence	(Song et al., 2017)
Cotton	*GhALKBH5*	Epigenetic (RNA m6A demethylation)	Modulates mRNA abundance of key flowering genes	Delays flowering when silenced	Controls dynamic photoperiod sensitivity	Functional evidence	(He et al., 2024)
Wheat	*Ppd-D1a*	2089-bp deletion in upstream promoter	Abolishes photoperiod sensitivity	Accelerated flowering under long-day conditions	Facilitates widespread introduction into high-latitude regions	Association & Functional evidence	(Beales et al., 2007)
Wheat	*Ppd-B1*	Copy number variation (Amplification)	Amplifies gene copies to increase dosage	Generates early-flowering day-neutral phenotype	Facilitates introduction into diverse photoperiod environments	Association evidence	(Díaz et al., 2012)
Wheat	*Vrn-A1*	Copy number variation (Amplification)	Amplifies gene copies to increase dosage	Strengthens vernalization requirement	Enhances adaptation to overwintering conditions at diverse latitudes	Association evidence	(Díaz et al., 2012)
Maize	*ZmCCT10*	Transposon insertion in promoter	Disrupts photoperiod-sensitive repressor binding	Facilitates floral initiation in long-day environments	Accelerates northward range expansion to high latitudes	Association & Functional evidence	(Li et al., 2023)

### 3.1 Reshaping photoperiod adaptation at the population point mutation level

Haplotypes derived from widespread single nucleotide polymorphisms (SNPs) serve as key genetic strategies for regulating flowering time and adapting to diverse latitudinal environments. GWAS have identified several superior haplotypes facilitating soybean latitudinal expansion, such as SOC1a, GmSGF14^H4^, and GmSTOP1a. Among these, a critical A/G single nucleotide polymorphism in the promoter region of *Tof18* (encoding *SOC1a*) is associated with the divergence of natural populations into distinct haplotypes with varying transcriptional activities and significant differences in flowering time. Specifically, the haplotype carrying the *Tof18*G allele confers an early-flowering phenotype, significantly enhancing soybean adaptability to high-latitude environments. Conversely, the *Tof18*A allele results in a late-flowering phenotype, which is advantageous for adaptation in low-latitude regions (Kou et al., 2022). Additionally, a frameshift mutation in the *GmSGF14^H^* haplotype disrupts its core 14-3–3 structural domain, resulting in a late-flowering phenotype that enhances soybean adaptability to specific latitudes (Jiang et al., 2023). Similarly, SNP analysis in cultivated rice revealed that key mutations in the *OsCCA1* coding region produce haplotypes (*OsCCA1a* and *OsCCA1d*) contributing to geographic adaptation. The *japonica OsCCA1a* haplotype promotes flowering, enabling expansion to higher latitudes, whereas the *indica OsCCA1d* haplotype delays flowering to ensure stable yields in low-latitude regions. This allelic variation in flowering time regulation represents a core strategy enabling major crops to adapt across a broad range of latitudes (Lee et al., 2022). Taken together, these findings underscore that point mutations have been identified across diverse species to mediate multidimensional crop expansion. Nevertheless, these loci have been predominantly characterized in a few model crops like soybean. Consequently, the functional conservation of these genes in other major species, such as cotton, rice, and wheat, warrants further investigation.

Population-level point mutations can also lead to the functional remodeling of key flowering integrator proteins. For instance, the photoperiod-insensitive soybean cultivar K3 harbors three quantitative trait loci (QTLs) associated with late flowering. Among them, a non-synonymous mutation in the fourth exon of *FT2a* suppresses its repressor function, resulting in the late-flowering phenotype of K3 under long-day and high-temperature conditions (Sun et al., 2019). Similarly, in cotton, a non-synonymous mutation was identified in the coding region of *GhTOFD06*, which is significantly associated with delayed flowering. Further analysis revealed that the homozygous wild-type C/C genotype underwent positive selection in cultivated populations. This allele attenuates the negative regulatory effect of *GhTOFD06* on flowering, thereby reducing photoperiod sensitivity and promoting early maturity (Xu et al., 2026). Collectively, these findings underscore that population-level point mutations provide a potential genetic basis for the adaptive evolution of flowering time in crops, enabling their accommodation to diverse regional environments. Despite these advances, population-level analyses of point mutations in cotton have been largely skewed towards fiber development and disease resistance, leaving the genetic basis of photoperiod adaptation poorly understood. Overall, these discoveries highlight point mutations as a pivotal genetic foundation for the adaptive evolution of flowering time, allowing crops to thrive in diverse agro-ecological zones. Future efforts to elucidate the functional conservation and divergence of loci driving latitudinal expansion across species will deepen our mechanistic understanding of photoperiodic adaptation and offer novel targets for accelerating crop expansion into new latitudes.

Notably, even when point mutations and sequence polymorphisms occur in non-coding regions, they can modulate crop traits by altering transcriptional activity. For example, a flowering time QTL on soybean chromosome 16 was identified as *FT5a*. The early-flowering (*ef*) and late-flowering (*lf*) alleles of this gene share identical coding sequences but harbor 15 DNA polymorphisms in the promoter, introns, and 3’UTR. These non-coding variations lead to significant differences in transcript abundance. Consequently, soybeans carrying this rare early-flowering haplotype can independently promote flowering without being regulated by the *E1* gene, providing a potentialgenetic basis for early-flowering adaptation in high-latitude regions (Takeshima et al., 2016).

### 3.2 Reshaping photoperiod adaptation at the level of chromatin modification

Beyond structural variations in DNA sequences, crops also employ epigenetic strategies, including DNA methylation and specific histone modifications, to precisely respond to photoperiod changes (Guarino et al., 2022; Shi et al., 2023). For instance, under optimal photoperiod conditions, the methylation level at CHH sites in the genome of short-day plants like rice is significantly suppressed, thereby activating the transcription of downstream key flowering genes (Wu et al., 2024). Furthermore, the rice methyl-CpG-binding domain protein OsMBD707 alters photoperiod sensitivity and flowering time by transcriptionally regulating flowering-related genes in the *Ehd1*-*Hd3a*/*RFT1* pathway (Qu et al., 2021). In cotton, a comparative analysis between *Gossypium hirsutum* (upland cotton) and wild cotton identified 519 differentially methylated genes (Song et al., 2017). Specifically, the *COL2D* gene is hypermethylated and silenced in wild cotton, conferring photoperiod sensitivity that prevents flowering during long-day summers. Conversely, demethylation-induced COL2D overexpression in domesticated cotton confers photoperiod insensitivity, potentially broadening its cultivation latitude and environmental resilience. These findings highlight the potential of epigenetic breeding in polyploidy. Furthermore, recent studies indicate that in early-maturing cotton varieties, DNA hypomethylation and elevated H3K9ac levels mediate the high expression of *GhAP1* compared to late-maturing varieties (Wang et al., 2023). Whether such differential methylation-driven changes in flowering time serve as key targets for the latitudinal expansion of cotton warrants further investigation.

Histone methylation-mediated transcriptional silencing also serves as a key driver of plant adaptation to complex environments. In soybean, the transcriptional repressor E1 directly binds to the promoter region of *GmMDE* and specifically elevates H3K27me3 levels at this locus. This epigenetically suppresses *GmMDE* expression, effectively blocking the positive feedback induction of downstream *GmFT2a/5a* and thereby precisely coordinating flowering time (Zhai et al., 2022). In rice, the abscisic acid (ABA)-responsive kinase SAPK8 directly phosphorylates the transcription factor ABF1; upon activation, ABF1 interacts with FIE2 to recruit the PRC2 complex to the target regions of the floral activators *Ehd1* and *Ehd2*. This markedly increases H3K27me3 deposition at these loci and represses their del transcription, ultimately aying flowering. These findings highlight a synergistic regulatory mechanism between hormone signaling and epigenetic modifications in plants (Tang et al., 2024). It will be an intriguing research direction to investigate whether differential methylation of these flowering genes mediates varietal adaptation across multiple varieties.

Notably, the epigenetic strategies adopted by crops during latitudinal adaptive expansion also extend to post-transcriptional RNA modifications. For instance, significant differences in N6-methyladenosine (m6A) modification levels have been observed between photoperiod-sensitive wild cotton and insensitive cultivated varieties, with wild types exhibiting significantly higher m6A levels under long-day conditions. Furthermore, silencing the m^6^A demethylase gene *GhALKBH5* reduces the mRNA abundance of multiple key flowering genes and causes delayed flowering, suggesting that dynamic m^6^A modification and demethylation of flowering-related transcripts may directly regulate photoperiod sensitivity in cotton (He et al., 2024). However, RNA modification-mediated differences in flowering have been confirmed in only a few species, and whether they drive crop expansion across different dimensions remains unknown. In summary, through a multilayered epigenetic regulatory network encompassing DNA methylation, histone methylation, and post-transcriptional RNA methylation (m^6^A), crops can adapt to complex photoperiod conditions across different latitudes without altering the underlying genomic sequence, thereby greatly facilitating their trans-regional geographical expansion. However, it is crucial to distinguish stable, heritable epigenetic variation from transient, environmentally induced epigenetic changes. In particular, epigenetic modifications that are developmental stage-specific or environmentally driven still require more rigorous functional validation across multiple generations and fluctuating environments to establish their causal role in latitudinal adaptation.

### 3.3 Evolutionary trajectories of photoperiodic adaptation driven by structural variations and copy number variation

Facing extreme long-day conditions at high latitudes or short-day environments at low latitudes, conventional point mutations often fail to rapidly disrupt the established photoperiodic balance. In such cases, genomic structural variations (SVs, including large deletions and transposon mobilization) and copy number variations (CNVs) often rapidly boost crop adaptation. Distinct from single nucleotide polymorphisms (SNPs), these large-scale variations can profoundly alter gene dosage, disrupt cis-regulatory elements to rewire gene expression networks, or modify local chromatin architecture. For example, naturally occurring CNVs can directly elevate the transcriptional abundance of flowering integrators via dosage effects, while transposon insertions often disrupt repressor binding sites, thereby rapidly rewiring the flowering regulatory network.

Structural variations (SVs) causing aberrant expression or inactivation of core flowering genes are frequently observed in crop evolution. For instance, the domestication and expansion from low-latitude to high-latitude regions involved the disruption of the photoperiod-sensitive flowering repressor *ZmCCT10* through a transposon insertion in its promoter region. This structural disruption reduced the photoperiod sensitivity, enabling flowering under long-day conditions and thereby accelerating its northward range expansion (Li et al., 2023). Similarly, in soybean, a Ty1-Copia-like retrotransposon (SORE-1) insertion within an intron of the *FT2a* gene confers allele-specific transcriptional repression, significantly reducing transcript abundance and consequently delaying flowering. This structural variant may facilitate soybean to maintain a prolonged vegetative growth phase under the short-day conditions at lower latitudes, thereby providing a genetic foundation for the further expansion of cultivation into even lower-latitude regions (Zhao et al., 2016).

Recent studies have identified a 24-bp InDel variant in the promoter of *GmSTOP1a* as a major genetic factor associated with soybean adaptation to acidic, low-phosphorus soils at low latitudes. Specifically, the haplotype carrying the 24-bp insertion is predominantly distributed in high-latitude soybean germplasm. This insertion disrupts the binding site for the transcription factor GmAGL12 on the *GmSTOP1a* promoter, thereby attenuating GmAGL12-mediated transcriptional activation of *GmSTOP1a*. In contrast, the 24-bp deletion allele that prevails in low-latitude soybean varieties maintains effective GmAGL12-mediated activation of *GmSTOP1a*, which in turn regulates the expression of multiple downstream genes involved in low-phosphorus tolerance, thus enhancing soybean fitness in acidic, phosphorus-deficient soils at low latitudes (Guo et al., 2026). Additionally, a 2089-bp deletion in the upstream promoter region of the wheat *Ppd-D1a* allele abolished photoperiod sensitivity and accelerated flowering under long-day conditions, thereby facilitating the widespread introduction of wheat into high-latitude regions (Beales et al., 2007).

Large-scale chromosomal structural variations (such as translocations or inversions) and cross-species large-fragment introgression are also core drivers in reshaping crop photoperiod sensitivity. Pan-genome analyses of upland cotton revealed a reciprocal translocation between A03 and A09 that delayed flowering in wild germplasm, while five large inversions (A06, A10, A12, A13, D03) broke photoperiodic constraints, conferring an extremely early-maturing phenotype. These structural variations provided the potential genetic basis for upland cotton’s expansion across diverse latitudes (Yang et al., 2026). In addition, approximately 18.16% of the upland cotton genome was introgressed from sea island cotton during domestication. This genome-wide segmental replacement enriched genetic diversity and introduced key early-maturing and high-latitude adaptive alleles, including the core photoperiod regulator *GhTOFD06*. This significantly advanced flowering time, thereby accelerating its global adaptation to high-latitude regions (Xu et al., 2026).

Gene copy number variation (CNV) has also played an indispensable role in the global expansion of polyploidy crops. Genetic analyses have demonstrated that multiple core genes regulating photoperiod response and vernalization requirements in the wheat genome, specifically *Ppd-B1* and *Vrn-A1*, have undergone significant copy number amplification and differentiation. For instance, alleles carrying increased *Ppd-B1* copy numbers have arisen independently at least twice during evolution, conferring a day-neutral phenotype that facilitated the introduction of wheat into diverse photoperiod environments. Concurrently, amplification of *Vrn-A1* copy number strengthens the vernalization requirement, further contributing to the adaptation of wheat to overwintering conditions across different latitudes (Díaz et al., 2012; Zikhali et al., 2017). In summary, the evolutionary expansion of crops across diverse global regions has not relied on a single genetic strategy. Instead, it has been driven by a multidimensional, synergistic regulatory network that integrates population-level point mutations, epigenetic modifications, and large-scale genomic variations. These genetic adaptive strategies not only enabled crops to overcome their original geographical and climatic constraints, thereby promoting their extensive worldwide cultivation, but also furnished a rich reservoir of natural genetic variation for early human agricultural domestication.

## 4 Discussion

Although substantial progress has been made in understanding photoperiod-driven crop adaptive evolution, crop expansion across natural environments is governed by a highly complex combination of environmental cues. To date, studies of crop adaptive evolution have concentrated mainly on the single dimension of photoperiodic signaling (González-Delgado et al., 2025). However, photoperiod is not the sole determinant of latitudinal adaptation; rather, it involves intricate genotype-by-environment (G×E) interactions with temperature, water availability, precipitation, and soil conditions (George et al., 2024; Jung et al., 2023). For instance, under specific high-latitude photoperiod regimes, adaptive alleles that are beneficial elsewhere may become maladaptive if they are incompatible with local thermal or altitudinal constraints. Therefore, future research requires a multidimensional analytical framework to accurately capture the multiple drivers of latitudinal expansion. Furthermore, while recent advances in pan-genomics and global genome-wide association studies (GWAS) have identified numerous alleles associated with regional expansion in crops such as soybean, rice, and maize (Naithani et al., 2023), a significant gap remains between these theoretical discoveries and their practical application in agriculture.”

During the course of natural domestication and modern breeding, shifts in photoperiod sensitivity rarely occurred in isolation but were often accompanied by concomitant variations in associated agronomic traits. For example, in soybean, transposon insertions and structural variations in *GmPHYA3* not only optimized flowering time but also conferred significant yield advantages under high-density planting at high latitudes (Qin et al., 2023). Likewise, a promoter InDel mutation in *GmSTOP1a* modulated photoperiod response and simultaneously enhanced tolerance to low-phosphorus conditions in low-latitude regions (Guo et al., 2026). Nevertheless, crop introduction is often accompanied by inherent physiological trade-offs. For example, expanding short-day plants into higher latitudes typically necessitates selecting for early flowering, which inherently curtails the vegetative growth phase and consequently compromises biomass accumulation and grain yield (Zhao et al., 2023a, 2023). Given these constraints, a critical imperative for future research is to leverage identified key expansion loci to accelerate regional adaptation without sacrificing established elite traits. To surmount such trade-offs, gene editing offers a promising avenue to break linkage drag. By precisely editing the coding or regulatory sequences of flanking deleterious alleles—through targeted knockouts or base substitutions—adverse effects can be eliminated without perturbing the target flowering genes. Furthermore, integrating these genetic interventions with intelligent decision support systems to dissect genotype-by-environment interactions will enable the development of breakthrough germplasms capable of achieving stable, synergistic enhancement of multiple traits across diverse agro-ecological zones. In addition, the synergy of AI-predicted genotype-environment alignments and synthetic biology-driven pathway assembly facilitates the introduction of *de novo* modules to drive the acquisition of superior traits.

In summary, the global expansion of crops represents more than a geographical redistribution of species; it is a broad biological process integrating environmental sensing, signal transduction, and developmental reprogramming. To confront the increasing challenges posed by extreme weather and rising food demand, modern breeding should employ advanced molecular design technologies to develop novel germplasm combining broad-spectrum environmental adaptability with high yield potential, thereby strengthening the basis for sustainable agricultural development and global food security.
